# Social participation and mental health of immunocompromised individuals before and after COVID-19 vaccination–Results of a longitudinal observational study over three time points

**DOI:** 10.3389/fpsyt.2022.1080106

**Published:** 2022-12-14

**Authors:** Gloria Heesen, Stephanie Heinemann, Frank Müller, Alexandra Dopfer-Jablonka, Marie Mikuteit, Jacqueline Niewolik, Frank Klawonn, Kai Vahldiek, Eva Hummers, Dominik Schröder

**Affiliations:** ^1^Department of General Practice, University Medical Center, Göttingen, Germany; ^2^Department of Rheumatology and Immunology, Hannover Medical School, Hanover, Germany; ^3^German Center for Infection Research (DZIF), Partner Site Hannover-Braunschweig, Brunswick, Germany; ^4^Department of Computer Science, Ostfalia University of Applied Sciences, Wolfenbüttel, Germany; ^5^Biostatistics Group, Helmholtz Centre for Infection Research, Brunswick, Germany

**Keywords:** SARS-CoV-2 vaccination, immunocompromised persons, social participation, mental health status, quality of life, observational study, longitudinal study, COVID-19

## Abstract

**Introduction:**

The coronavirus disease 2019 (COVID-19) pandemic impacted how people perform their daily lives in manifold and sometimes massive ways. Particularly, individuals who are at high risk for a severe disease progression, like immunocompromised people, may have experienced drastic changes in social participation during the pandemic. A COVID-19 basic vaccination may have changed the safety behavior of immunocompromised individuals in terms of infection risk and thereby influence social participation and mental wellbeing.

**Methods:**

This study aims to investigate self-perceived social participation at baseline before and at follow-up 1 and 6 months after basic vaccination. Beginning in March 2021, 274 immunocompromised persons 18 years or older were enrolled in the COVID-19 Contact Immune study (CoCo study) in Lower Saxony, Germany. Measurements were performed at three time points regarding social participation [Index for the Assessment of Health Impairments (IMET)], mental health [Patient Health Questionnaire-4 (PHQ-4)], subjective health status (five-point Likert-scale) and quality of life (five-point Likert-scale).

**Results:**

In total, 126 participants were included in the final analysis. About 60% of the participants showed increasing social participation over time. The greatest increase in social participation was observed within the first month after basic vaccination (*p* < 0.001). During the following 5 months, social participation remained stable. The domains “social activities,” “recreation and leisure” and “close personal relationships” were responsible for the overall change in social participation. No association was found between social participation and mental health, sociodemographic or medical factors (except hypertension).

**Discussion:**

It is unclear why social participation increased after basic vaccination. Perceived vaccine efficacy and a feeling of being protected by the vaccine may have caused relaxed social distancing behaviors. Reducing safety behaviors may, however, increase the risk of a COVID-19 infection for immunocompromised individuals. Further investigations are needed to explore the health-related consequences of more social participation among immunocompromised persons.

## 1 Introduction

Since the emergence of severe acute respiratory syndrome coronavirus type 2 (SARS-CoV-2) in Wuhan in December 2019 and the global spread of the virus, the pandemic has greatly changed daily lives and social participation (1–3). To minimize new infections, all social events and contacts outside the household were restricted. The German population was asked to practice social distancing, to observe hygiene regulations and to wear a mask. There were several “lockdowns” in Germany, the first starting 22nd March 2020 for 3 weeks and the last one from 16th December 2020 for about 4 weeks (4). The population subsequently spent a larger part of the day at home with less contact to others (1). The above-mentioned circumstances could have a negative impact on social participation, which in turn is negatively associated with health status and quality of life (5, 6).

The impact of COVID-19 related lockdowns on lifestyle habits and behavioral risk factors cannot be ignored. Immunocompromised individuals as a vulnerable population are particularly at risk for severe COVID-19 and therefore also at a higher risk for psychological distress (7). Also non-pharmacological treatments for mental disorder such as enhancing exercise were restricted during the pandemic (8, 9). Nürnberger et al. reported that immunocompromised individuals as well as women in general have a high level of COVID-19 anxiety (10). For this reason, it is likely that these individuals follow measures more diligently compared to non-immunocompromised individuals or they take additional infection prevention measures even beyond the official requirements. Additionally, family and friends of such persons could be more careful in keeping their distance to avoid the risk of infecting vulnerable loved ones.

Stipulating infection prevention measures that require individuals to reduce social contacts create a difficult tradeoff between physical and mental health. Initial studies confirm this psychological burden (11, 12).

The COVID-19 vaccination is considered the most effective protective measure to prevent severe courses of COVID-19. Recommendations on the number and timing of vaccine doses changed frequently during the pandemic. At the beginning of our study, it was assumed that a basic vaccination was achieved 14 days after the second vaccination. All in Germany licensed COVID-19 vaccines are highly effective in protection against severe and lethal COVID-19 (13–16). At the time of the first vaccinations in 2021, it was assumed that social restrictions could be eased after achieving a high vaccination rate about 80% (17). Today, even higher vaccination rates and booster vaccinations are known to be necessary in presence of highly infective and partly immune-escape SARS-CoV-2 virus variants (18).

Vaccine efficacy in immunosuppressed people remains unclear (19, 20). Due to the new virus variants of concern, vaccine effectiveness has decreased even for immunocompetent persons, in particular against infection and any COVID-19 disease, whilst effectiveness against severe disease remains high (21, 22). Factors influencing social participation could be the number and type of immunosuppressive medications and comorbidities as well as self-perceived vulnerability for a severe COVID-19 course. Due to viral variants of concern, the difficult predictability of the individual case and studies with only small numbers of investigated cases, the vaccine effectiveness for immunocompromised persons remains not entirely clear. Nevertheless, vaccination status may change the perceived importance of safety behaviors and decrease the compliance with measures to limit the spread of the disease. Social participation could increase because of a sense of security, even though the actual level of protection remains uncertain.

Understanding the impact of vaccination on social participation and mental health during the pandemic period will lead to increased understanding of the impact of infection prevention measures upon everyday life and health. This knowledge can be used when planning further measures.

The main hypotheses tested in this study are:

(1) Social participation improves after basic vaccination against COVID-19 and remains improved or continues to improve after 6 months.
(2) Changes in mental health are associated with changes in social participation.
(3) Sociodemographic, medical or pandemic-related factors are associated with changes in social participation.

## 2 Materials and methods

### 2.1 Research design and participants

The COVID-19 Contact Immune study (CoCo study) is a longitudinal, prospective, observational study and was conducted at two large university hospitals in Göttingen and Hannover in Germany (23). Beginning in March 2021, we recruited persons who (1) were 18 years or older, (2) able to provide informed consent and (3) immunocompromised due to an immunosuppressive drug therapy. Exclusion criteria were (1) refusal/inability to provide informed consent or (2) contraindications to blood testing. There were no further inclusion or exclusion criteria.

We recruited study participants with newspaper advertisements and posters in hospitals, vaccination centers, and in offices of rheumatologists. Due to vulnerability of immunocompromised people, we organized the study so that participation was possible with very little in-person contact. Interested persons contacted the study center by phone or e-mail. The declaration of consent by the participants could be given by telephone, videocall, or in person during a short interview for information and enrollment purposes. The study team encouraged every participant to comply with all publicly recommended measures and regulations. The signed consent form was returned by mail. The study team send the study materials (e.g., questionnaires and blood sample kits) to recruited participants by mail at the start of the study. Pencil and paper questionnaires assessed the social participation and mental health at three different time points: at enrollment before basic vaccination against COVID-19 (T0) and 1 month (T1) and 6 months (T2) after basic vaccination. There was an additional computer-assisted telephone follow-up from December 2021 until January 2022 to determine if and when the participants got a third vaccine dose. Further information about the CoCo study can be gathered in the study protocol (23).

### 2.2 Measures

#### 2.2.1 IMET

The Index for the Assessment of Health Impairments (IMET) is based on the International Classification of Functioning, Disability, and Health (24). It was initially developed to collect data about social participation in rehabilitation research. The questionnaire measures if the persons perceive any impairments regarding nine dimensions of their social participation using a 11 level Likert-scale (0–10). Higher scores indicate a greater impairment. The sum score of these nine items describes the overall social participation with a high internal reliability (Cronbach’s alpha of 0.90). This instrument was already used during the COVID-19 pandemic by Mergel and Schützwohl to define impairments of social participation in people without and with mental disorders (25). A between group change of 4.41 points between the intervention and control group of a rehabilitation intervention was observed by Hüppe et al. (26).

#### 2.2.2 PHQ-4

The Patient Health Questionnaire-4 (PHQ-4) is an ultra-short questionnaire, consisting of two items collect data about depression (PHQ-2) and two items measure anxiety (GAD-2, Generalized Anxiety Disorder Scale-2). It is a reliable and validated questionnaire that uses four-point Likert scales. Sum scores from 0 to 12 are achievable with higher scores indicating worse psychological health. The specificity of the PHQ-4 is 94.5% and the sensitivity 51.6% at a cutoff of 6 (27). The PHQ-4 has been used in other studies to evaluate mental health during the COVID-19-pandemic (28).

### 2.3 Sample size calculation

The CoCo study explores the effect of the COVID-19 vaccination on social participation. In Germany, the first COVID-19 vaccine was authorized on December 27, 2020. The COVID-19 CoCo study was registered on the December 30, 2020. Until this date, no study had investigated social participation before and after a COVID-19 vaccination. Therefore, no sample size calculation was done prior to this study. We assume a medium effect size (Cohen’s *f* = 0.25) on social participation, measured by the IMET before and after vaccination. Using G Power for sample size calculation, 44 participants would be sufficient with a correlation of 0.5 among the repeated measures and an alpha level of 5% to detect such an effect. Using non-parametric tests, a 15% higher sample size is needed, resulting in 51 participants.

### 2.4 Statistical analysis

Participants were excluded from statistical analysis if they (a) did not state their immunosuppressive medication or underlying disease, (b) already had a basic COVID-19 vaccination at baseline (14 days or more after two vaccinations or after one in case if the COVID-19 Vaccine Janssen was used), (c) the first and the second questionnaire were completed within an interval of 21 days or less, or (d) participants did not complete the whole IMET questionnaire at all three time points. Sociodemographic and medical are reported with number of participants in each category and the corresponding proportion. Age is reported with mean and standard deviation (SD). For a sensitivity analyses, the participants were divided into two groups: one group in which social participation improved and one group in which social participation remained stable or worsened between T0 (before vaccination) and T2 (6 months after basic vaccination). Between these groups, the sociodemographic (gender, age, school education, city resident size, household) and medical factors (underlying disease, comorbidities, degree of disability, immunosuppression medication, number of immunosuppressants, therapy paused for COVID-19 vaccination) were compared using the Fisher-Exact test for 2 × 2 contingency tables and the Freeman-Halton extension for larger tables. Median scores with the corresponding interquartile range (IQR) for all included scales and subscales for the three observation points are reported and compared between the three time points using the Quade test because of a non-normal distribution of the data. When testing subscales, the *p*-value was adjusted using the Bonferroni method. Participants were grouped according to their IMET courses (increased, stable, or decreased social impairments) between T0–T1 and T1–T2 resulting in nine possible courses (Supplementary material). Stable social impairment was defined as a maximum IMET sum score difference of one point between two time points.

To test for an association between the third vaccine dose and a change of the IMET score between the timepoints T1 (1 month after basic vaccination) and T2 (6 months after basic vaccination), the Kruskal-Wallis test was performed. Participants without information about the third vaccination were excluded. The IMET scores at baseline were compared to mean scores of persons with an inflammatory bowel disease from 2014 (6) in order to categorize our sample into two groups of participants with lower or higher social participation compared to pre-pandemic levels.

The association between mental health and social participation was tested using repeated measure correlation and McNemar’s test for categorized PHQ-4 scores (cutoff score six or higher). Additionally, we compared the incidence of mental health disorders using the PHQ-4 in participants with an increased or decreased/stable social participation. For this analysis, participants with a PHQ-4 score above the cut-off at baseline or with a current self-stated depression were excluded.

Data regarding the COVID-19 incidence in the Göttingen county and the hospitalization rate of COVID-19 cases in Germany were gathered from the Website of Germany’s public health institute (29).

All statistical analyses and graphical illustrations were performed using R 4.1.1. Stats (30), rmcorr (31), and ggplot2 (32).

## 3 Results

The first participant was included on March 28, 2021. The last participant was included in May 20, 2021. No participant completed the survey during the period of national lockdown in Germany.

**FIGURE 1:**
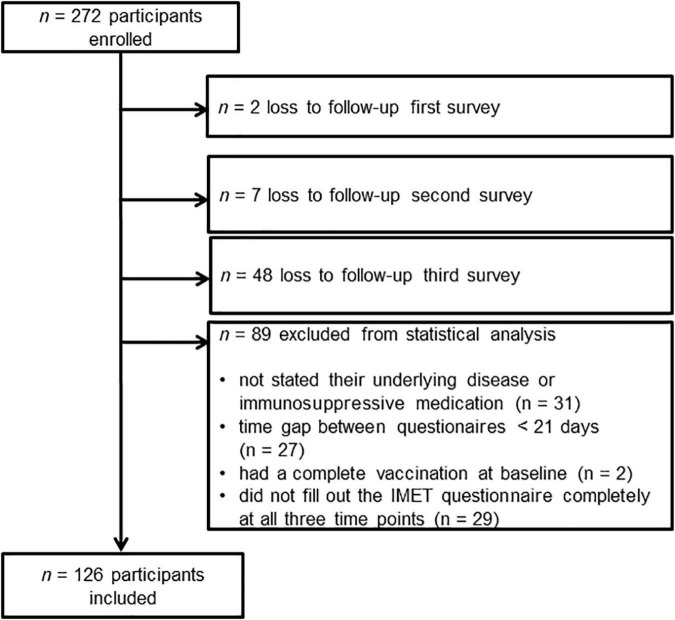
Flowchart of participant inclusion and exclusion.

After applying the in- and exclusion criteria, 126 participants were included in the final analysis (see Figure 1). The mean difference between T0 and T1 was 81.6 days (SD: 22.5) and between T0 and T2, 236.5 days (SD: 27.7).

### 3.1 Participants’ characteristics

The majority of participants (70.4%) were female. The mean age was 52.1 years (SD: 13.0). Most (59.5%) had completed high school. Most participants lived in rural areas (41.3%), followed by 26.5% who lived in cities with more than 100,000 inhabitants (Table 1).

**TABLE 1:** Sociodemographic, medical, and pandemic-related characteristics of study participants with increased social participation and participants with decreased or stable social participation.

	All (*N* = 126) *n* (%)	SP increased (*N* = 78) *n* (%)	SP decreased or stayed stable (*N* = 48) *n* (%)	*p*
**Gender**				
Male	37 (29.6)	24 (31.2)	13 (27.1)	0.81
Female	88 (70.4)	53 (68.8)	35 (72.9)	
**Age, years [mean (SD)]**	52.1 (13.0)	50.7 (13.8)	54.4 (11.4)	
<40	28 (22.4)	21 (27.3)	7 (14.6)	0.27
40–65	76 (60.8)	45 (58.4)	31 (64.6)	
>65	21 (16.8)	11 (14.3)	10 (20.8)	
**School education**				
Low	8 (6.3)	3 (4.1)	5 (10.6)	0.22
Middle	35 (27.8)	18 (24.3)	17 (36.2)	
High	75 (59.5)	51 (68.9)	24 (51.1)	
Not specified	3 (2.4)	2 (2.7)	1 (2.1)	
**City resident size**				
<5,000	50 (41.3)	32 (41.0)	18 (37.5)	0.65
5,000–20,000	28 (23.1)	14 (18.0)	14 (29.2)	
20,000–100,000	11 (9.1)	7 (9.0)	4 (8.3)	
>100,000	32 (26.5)	21 (26.9)	11 (22.9)	
Household*				
Parenting	26 (20.6)	19 (24.4)	7 (14.6)	0.26
Single parent	2 (1.6)	2 (2.6)	0 (0.0)	0.53
Living alone	22 (17.5)	14 (18.0)	8 (16.7)	1
Care of relatives other than children	16 (12.7)	9 (11.5)	7 (14.6)	0.78
Underlying disease*				
Rheumatological disease	49 (38.9)	30 (38.5)	19 (39.6)	1
Inflammatory bowel disease	21 (16.7)	14 (18.0)	7 (14.6)	0.81
Psoriasis	19 (15.1)	11 (14.1)	8 (16.7)	0.80
Multiple sclerosis	18 (14.3)	14 (18.0)	4 (8.3)	0.19
Solid organ transplant	10 (7.9)	6 (7.7)	4 (8.3)	1
Other	12 (9.5)	4 (5.1)	8 (16.7)	0.051
Comorbidities*				
Hypertension	53 (42.1)	25 (32.1)	28 (58.3)	0.01
Diabetes type 2	6 (4.8)	6 (7.7)	0 (0.0)	0.08
Depression	15 (11.9)	11 (14.1)	4 (8.3)	0.41
Severe obesity	17 (13.5)	11 (14.1)	6 (12.5)	1
Renal insufficiency	9 (7.1)	6 (7.7)	3 (6.3)	1
Chronic pain	23 (18.2)	15 (19.2)	8 (16.7)	0.81
Asthma bronchiale	12 (9.5)	7 (9.0)	5 (10.4)	0.77
Allergies	27 (21.4)	17 (21.8)	10 (20.8)	1
**Formal degree of disability (%)^1^**				
No impairment (0)	48 (38.4)	31 (39.7)	17 (35.4)	0.86
Low impairment (20–49)	23 (18.4)	15 (19.2)	8 (16.7)	
Moderate impairment (50–74)	43 (34.4)	25 (32.1)	18 (37.5)	
Severe impairment (75–100)	11 (8.8)	6 (7.7)	5 (10.4)	
Immunosuppressive medication*				
Glucocorticosteroids	44 (34.9)	29 (37.2)	15 (31.3)	0.57
Methotrexat	30 (23.8)	20 (25.6)	10 (20.8)	0.67
TNF inhibitor	26 (20.6)	14 (18.0)	12 (25.0)	0.37
Azathioprin	10 (7.9)	6 (7.7)	4 (8.3)	1
Tacrolimus and everolimus	9 (7.1)	5 (6.4)	4 (8.3)	0.73
Others	34 (27.0)	21 (26.9)	13 (27.1)	1
Therapy paused for COVID-19 vaccination	30 (23.8)	16 (20.5)	14 (29.2)	0.24
**Number of immunosuppressant substances taken**				
1	73 (57.9)	41 (52.6)	32 (66.7)	0.3
2	41 (32.5)	28 (35.9)	13 (27.1)	
3 or more	12 (9.5)	9 (11.5)	3 (6.3)	
**Booster vaccination**				
Before T2	46 (36.5)	31 (39.8)	15 (31.3)	0.51
After T2	57 (45.2)	32 (41.0)	25 (52.1)	
Unknown	23 (18.3)	15 (19.2)	8 (16.7)	

*Multiple selection possible. ^1^Based on the German social law measuring physical, mental, and social impairment.

The most frequent underlying diseases were rheumatological diseases (38.9%), inflammatory bowel diseases (16.7%), psoriasis (15.1%), and multiple sclerosis (14.3%). Two or more immunosuppressive medications were taken by 42% of the participants. Oral Corticosteroids and Methotrexate (MTX) were the most frequently taken medications. About one-quarter of the participants (23.8%) interrupted their immunosuppressive therapy for the COVID-19 vaccination. Hypertension was the most common comorbidity with 42.1%. About 60% of participants had a formally recognized degree of disability.

At the second follow-up, 36.5% of participants had already received a third vaccine dose, 45.2% had only received basic vaccination and 18.3% did not state this information.

Hypertension was the only sociodemographic, medical or pandemic-related variable which differed significantly between participants with increased (62.7%) and participants with decreased or stable (37.3%) social participation (*p* = 0.01).

### 3.2 Measured changes over three timepoints

A significant change between the three timepoints was only observed in the IMET score (see Table 2). *Post hoc* analysis revealed a significant decrease of the IMET scores between T0 and T1 (*p* = 0.01) and T0 and T2 (*p* = 0.001). The change between T1 and T2 was not significant. After Bonferroni correction of the *p*-values, the domains “recreation and leisure,” “social activities,” and “close personal relationships” showed a significant change. The highest change was observed in the domain “social activities.” Over 50% of the participants reported no restrictions in the domain “usual activities of daily life” in all three time points. Identical results could also be obtained when excluding participants with not specified or a low level of school education, single parents, and participants with not specified underlying diseases (*n* = 101).

**TABLE 2:** Median scores and interquartile range of included measures for each observed time point.

Median (IQR)	T0	T1	T2	*F*
IMET score (*N* = 126)	**32 (24.75)**	**23.5 (26.75)**	**22 (25)**	**7.42**
Usual activities of daily life^5^	0 (2)	0 (2)	0 (2)	0.60
Family and domestic responsibilities^6^	1 (3)	1 (3.75)	1.5 (3.75)	1.32
Getting things done outside of home^4*^	3 (5)	2 (3)	2 (3.75)	4.49
Daily tasks and obligations^8^	2 (4.75)	2 (4)	2 (4)	0.84
Recreation and leisure^9^	**6 (6.5)**	**3 (5)**	**3 (4)**	**18.15**
Social activities^9^	**9 (5)**	**5 (5)**	**4 (6)**	**30.73**
Close personal relationships^7^	**2 (6)**	**2 (4)**	**1.5 (5)**	**4.91**
Sex life^7^	2 (5)	3 (6)	2 (6)	2.30
Stress and extraordinary strain^2^	3 (4.75)	2 (4)	3 (5)	2.30
PHQ-4 (*N* = 122)	2.5 (3)	2 (3)	3 (3)	1.39
GAD-2 (*N* = 124)*	1 (2)	1 (2)	1 (2)	3.25
PHQ-2 (*N* = 122)	2 (1)	1 (1)	1 (3)	1.16
QoL (*N* = 126)	3 (2)	3 (2)	3 (2)	1.65
Health status (*N* = 126)	3 (2)	3 (2)	3 (2)	0.75

Bold: significant change between one of other time points using Quade test. *P < 0.05 but not significant after Bonferroni correction. IMET, Index for the Assessment of Health Impairments; PHQ-4, Patient Health Questionnaire-4; PHQ-2, Patient Health Questionnaire-2; GAD-2, Generalized Anxiety Disorder Scale-2. Superscript indicating targeted ICF domain.

### 3.3 Social participation courses

To give a better insight into the courses of social participation, we describe each possible combination of the change of social participation in Table 3. Furthermore, these courses are shown in the form of a graph in Supplementary Figure 2. One of every six participants (17.5%) showed fewer limitations in their social participation between T0 and T1 as well as between T1 and T2. The median IMET score decreased in this group by 23 points. The majority of all participants, 34.1% experienced fewer limitations in their social participation 1 month after the second COVID-19-vaccination and in the following 5 months the number of limitations to social participation slightly increased again. Nevertheless, compared with their baseline scores, the limitations to social participation decreased in total by a median of five points.

**TABLE 3:** Index for the Assessment of Health Impairments (IMET) score courses between three timepoints and the corresponding median of IMET score for a particular course in parentheses.

	T1 – T2			
		Fewer limitation	No change	More limitations
**T0 – T1**	**Fewer limitations**	17.5 (−23)	7.1 (−9)	34.1 (−5)
	No change	4.0 (−21)	0 (–)	5.6 (6)
	More limitation	23.0 (1)	2.4 (7)	6.4 (21)

Data % of all participants (median IMET scores difference T2–T0 in each cell). Cells with gray filling: fewer limitations in social participation (decreased IMET scores).

About one-third of the participants showed an increased amount of limitations to social participation between T0 and T2. About one-fourth of all participants (23%) experienced more limitations 1 month after basic vaccination and fewer limitations after 6 months; the median IMET score increased by one point.

58.7% of all participants had fewer limitations to social participation 1 month after basic vaccination. About 10% had a stable social participation and 31.8% experienced more limitations. Of those who experienced more limitations, 20% continued this trend in the next 5 months. Of those who improved (i.e., experienced fewer limitations), about 30% improved further in the next 5 months. The amount of change can be seen in Table 3.

Six months after basic COVID-19-vaccination, 62.6% reported fewer limitations (gray-colored cells in Table 3) and 37.4% more limitations to social participation. None of the participants remained the same, defined as plus/minus one point on the IMET score after 6 months.

### 3.4 Association between social participation and mental health

Repeated correlation measures show a small correlation of IMET and PHQ-4 scores comparing the baseline and follow-up time points [*r* = 0.17, 95% CI (0.05; 0.29)].

The incidence of severe mental health symptoms measured by the PHQ-4 did not change during the time points using the McNemar’s test. The incidence of mental disorders between T0 and T2 did not differ significantly between participants with an increased social participation (7 out of 62) and participants with a stable *t* or decreasing social participation (5 out of 36). Only those participants with a PHQ-4 score below the cut-off point (<6) or without a self-stated depression at T0 (*N* = 98) were included in this analysis.

### 3.5 Possible confounding pandemic factors

**FIGURE 2:**
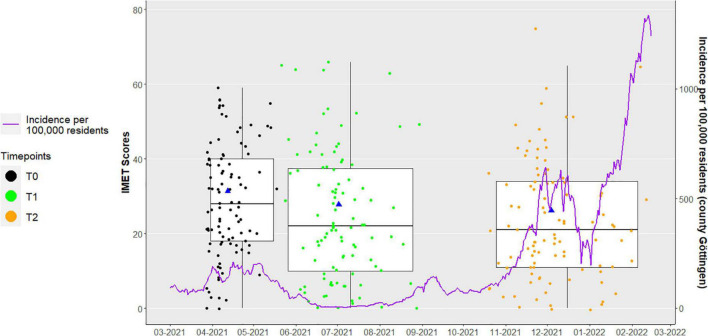
Median and mean change of social participation between T0, T1, and T2. Blue triangle: mean Index for the Assessment of Health Impairments (IMET) scores and mean date for each observation point; purple line: secondary Y-Axis [Incidence per 100,000 residents (county Göttingen)].

Figure 2 shows a higher incidence of COVID-19 cases per 100,000 inhabitants at timepoint T0 than at T1. At T2, the incidence is even higher than at T0. With regard to the levels of hospitalized COVID-19 cases in Germany, a similar course can be observed (Supplementary Figure 1). The IMET median scores become lower over all three timepoints, indicating fewer limitations to social participation. No association between the incidence and the limitations of social participation can be seen. These findings can be confirmed by repeated measure correlation [*r* = −0.03 95% CI. (−0.16; 0.09)].

The distribution between participants who improved between T1 and T2 and participants who worsened or remained the same did not differ when stratified by third vaccine dose before or after T2 (*p* = 0.84). The Wilcoxon test comparing IMET score differences (T0–T2) between those who had a third vaccine dose before and those who had their third vaccination after T2 showed no significant difference (*p* = 0.45).

Those participants whose IMET score at baseline was higher (i.e., more limitations to social participation) compared to pre-pandemic conditions were more likely to improve (67.4%) until T2 than those whose IMET score was lower or at norm (45.2%) (*p* = 0.03). Using the Wilcoxon test, this finding was also confirmed (*p* < 0.001).

## 4 Discussion

This study focuses on the change in social participation between three time points before a basic COVID-19 vaccination to 6 months after vaccination. About 60% of our participants had an increased social participation (i.e., experienced fewer limitations to social participation) at median about 10 IMET points and 40% had a decreased social participation 6 months after basic COVID-19 vaccination. Sociodemographic and medical factors (except hypertension) did not differ between these two subgroups. The results of this study support the hypothesis that a basic vaccination is associated with increased social participation. The reduction of limitations to social participation may motivate individuals to get vaccinated and consequently increase the vaccination rate. Between 1 and 6 months after the basic vaccination, the level of social participation remains stable. The domains “recreation and leisure,” “social activities,” and “close personal relationships” were mainly responsible for the reduction of limitations to social participation, whereby “social activities” was the area where limitations decreased most.

The courses of social participation were different within 6 months of basic vaccination. More than the half of the participants had a heterogeneous course. About one-fifth of the participants showed an increase of social participation over all three time points. Nearly one-quarter of all participants reported more limitations 1 month after a full vaccination and fewer limitations 5 months later. With regards to this up and down profile, it is important to note that the actual protection provided by the COVID-19 vaccination is still unclear for immunocompromised individuals in general and even more so on an individual level. A possible explanation for the reduction of limitations at T2 is the fact that participants of the CoCo immune study could see the results of their T1 antibody-test online about 4 weeks after T1. Therefore, at T2, the participants knew if they had developed antibodies against SARS-CoV-2. This may have decreased their sense of vulnerability and improved their social participation–although the study personnel emphasized that the development of antibodies is not synonymous with protection and all participants were advised to continue to observe safety measures. There was no association between social participation and a third vaccination, local COVID-19 incidence rates, or mental health. Those participants whose social participation levels were worse compared to pre-pandemic norm values before a basic COVID-19-vaccination improved more frequently than those whose social participation was better or at the norm.

Most studies investigating social participation among certain COVID-19 risk groups did so in a cross-sectional design at one time point (33–35). Mergel and Schützwohl also observed increased impairments in social participation by using the IMET questionnaire during the first year of the COVID-19 pandemic, when no COVID-19 vaccination was available (25). The median amount of change (10 points) is similar to the change in social participation in a study which investigated the impact of a rehabilitation on the social participation of people with chronic inflammatory bowel diseases (26).

This study did not indicate an association between the change in social participation and several sociodemographic factors. In other studies, the elderly are often described as a vulnerable group with less social participation and less mental health during the pandemic (36, 37). In our study, there was no significant impact of age on the change in social participation. This may be explained by the fact that this study only examined the changes of social participation and did not consider the baseline social participation level. Age-related differences may be masked by immunosuppressive medication, as a greater impact of this on social participation is possible.

Gender influenced participation in work life during the COVID-19-pandemic according to Flor et al. Women were more often affected by losing their jobs or dropping out of school (38). Another study found that living in small villages is negatively associated with social participation (39). These findings were not confirmed by our study of participants with a severe chronic illness and immunosuppressive treatment.

Hypertension was the only comorbidity that had a significant influence on the change in social participation. Participants with hypertension were more likely to experience more limitations to their social participation. Deng et al. observed that participants with hypertension had a nearly 4.5-fold higher risk of death when infected with SARS-CoV-2. It seems possible that participants with hypertension have a greater fear of a severe COVID-19 course and therefore restrict themselves to an above-average extent (40). However, the impact of hypertension on social participation or potential confounders need to be investigated further.

Our results do not prove the assumption that mental health is related with changes in social participation. In contrast to our findings, higher incidence rates of depressive symptoms were observed in older people with less social participation during the COVID-19 pandemic by Noguchi et al. (36). This difference can be explained by different participant groups and study settings. Other studies investigated the anxiety and depression levels at one timepoint after a COVID-19 vaccination that improved already after one vaccination dose (41).

Changes in social participation were not correlated to changes in COVID-19 incidence rates. One explanation for this lack of a pandemic-related effect could be that over the course of the pandemic, people got more familiar with digital communication and an accustomed to the pandemic realities (42). Becoming accustomed to the pandemic influences wellbeing and social participation as well (43). The temporary mitigation of the restrictions during the study period could have enabled participants to improve their social participation. Infections with the omicron variant lead to less severe courses of illness and less hospitalizations compared to earlier variants, and in general there were less severe or fatal cases than in the earlier days of the pandemic. Likely due to increasing vaccination levels. Therefore, the threat of a severe COVID-19 course during the omicron phase of the pandemic was likely perceived as lower than in the earlier measurements (44, 45). A common “pandemic fatigue” or getting more used to the situation could have influenced results at the follow-up timepoints.

The COVID-19-vaccination effectiveness in immunocompromised people depends on the underlying condition, immunosuppressant medication, and general health and is therefore variable (46). The currently prevailing omicron variant is less severe but still worrying and a threat to all individuals, especially for immunocompromised people. Loosening safety measures exposes to a high risk of infection (47).

This study has several limitations. Out of 272 recruited participants 126 were included in the final analysis. Due to the recruitment strategy and the loss-to-follow-up the sample of this study is not representative for all immunocompromised people in Germany. We were unable to reach all study participants in our telephone follow-up to gather the information about the third vaccine dose. Also, our sample was recruited as a non-random convenience sample which could lead to a selection bias. However, the CoCo Immune Study represents a true-to-life cross-section of primary care reality.

There is no control group of immunosuppressed individuals who refused COVID vaccination, nor a healthy control group. Such a control group would increase the validity of our results regarding the influence of the COVID-19 vaccination on social participation in immunocompromised people and helped to differentiate disease specific effects from generic phenomena. However, our ethics committee advised against requesting people at high risk for a severe COVID-19 course to stay unvaccinated for 6 months due to the health risks for both participants themselves and others. Sociodemographic and disease-related factors were collected only with the baseline questionnaire. Therefore, the underlying different chronic diseases of the participants may have worsened over the study due to normal chronic disease progression.

There are several factors beside the COVID-19 vaccination that could have confounded our results. The pandemic situation with its measures and restrictions is very quickly changing. The incidence rates of one county may not be sufficiently correlated to the whole pandemic situation. Since the participants’ exact place of residence is unknown due to data protection measures, it was impossible to pinpoint the exact local COVID incidence rate for each participants’ residential community. Even so, this study is located in the county of Göttingen and most participants come from the greater Göttingen region. The incidence rates in Göttingen, which were similar to the overall incidence rates in Germany though on a usually somewhat lower level. Limitations to social participation could be influenced by changing seasons. Colder outdoor temperatures lead to a higher incidence of contagious diseases other than COVID-19, for which immunosuppressive people are at a high risk. Therefore, perceived social participation could be influenced by the observational timepoint in our study. Due to the long duration of this study, this bias could be minimized. The IMET scale reflects the participants’ perceived impairments and not their actual social activities (i.e., how limited the participant feels but not the number of social interactions themselves). The questionnaire was not developed to reflect social participation during a pandemic situation. An improved questionnaire which takes particular pandemic situations into account may lead to more valid results.

## 5 Conclusion

One month after basic COVID-19 vaccination, social participation increased and then stayed stable for the next 5 months. Social activities, recreation and leisure, and close personal relationships were mainly responsible for changes in social participation following COVID-19 vaccination. Our sample shows a high variability between the individual participants. The protection expected of a COVID-19 vaccination is likely to have increased social participation levels. On the one hand, individual vaccine effectiveness in this particular group is quite unpredictable, and increased social participation comes with a higher risk of infection for immunocompromised individuals, for example, due to less safety behavior. On the other hand, improved social participation may be a convincing argument to motivate immunocompromised people to get vaccinated. These results highlight the heterogeneity of changes in social participation in a homogeneous group during a similar time period. In addition, other factors that might influence social participation need to be investigated in order to target vulnerable groups and plan interventions. Therefore, studies with larger sample sizes or qualitative studies might be useful.

## Data availability statement

The raw data supporting the conclusions of this article will be made available by the authors, without undue reservation.

## Ethics statement

The studies involving human participants were reviewed and approved by the Institutional Review Board of Hannover Medical School (9948_BO_K_2021) and University Medical Center Göttingen (29/3/21). The patients/participants provided their written informed consent to participate in this study.

## Author contributions

GH and FM: conceptualization. DS and GH: methodology and writing – original draft preparation. DS: formal analysis and visualization. GH, SH, and FM: investigation. SH, FM, MM, EH, FK, JN, KV, and MM: writing – review and editing. FM, SH, and EH: funding acquisition. All authors have read and agreed to the published version of the manuscript.
