# Supplementary material for: Social participation and mental health of immunocompromised individuals before and after COVID-19 vaccination–Results of a longitudinal observational study over three time points
Source: Front Psychiatry. 2022 Dec 14;13:1080106. doi: 10.3389/fpsyt.2022.1080106 (PMC9795223; doi:10.3389/fpsyt.2022.1080106)
Supplement: Supplementary file 1 [file Data_Sheet_1.docx]

**SUPPLEMENTARY MATERIAL**


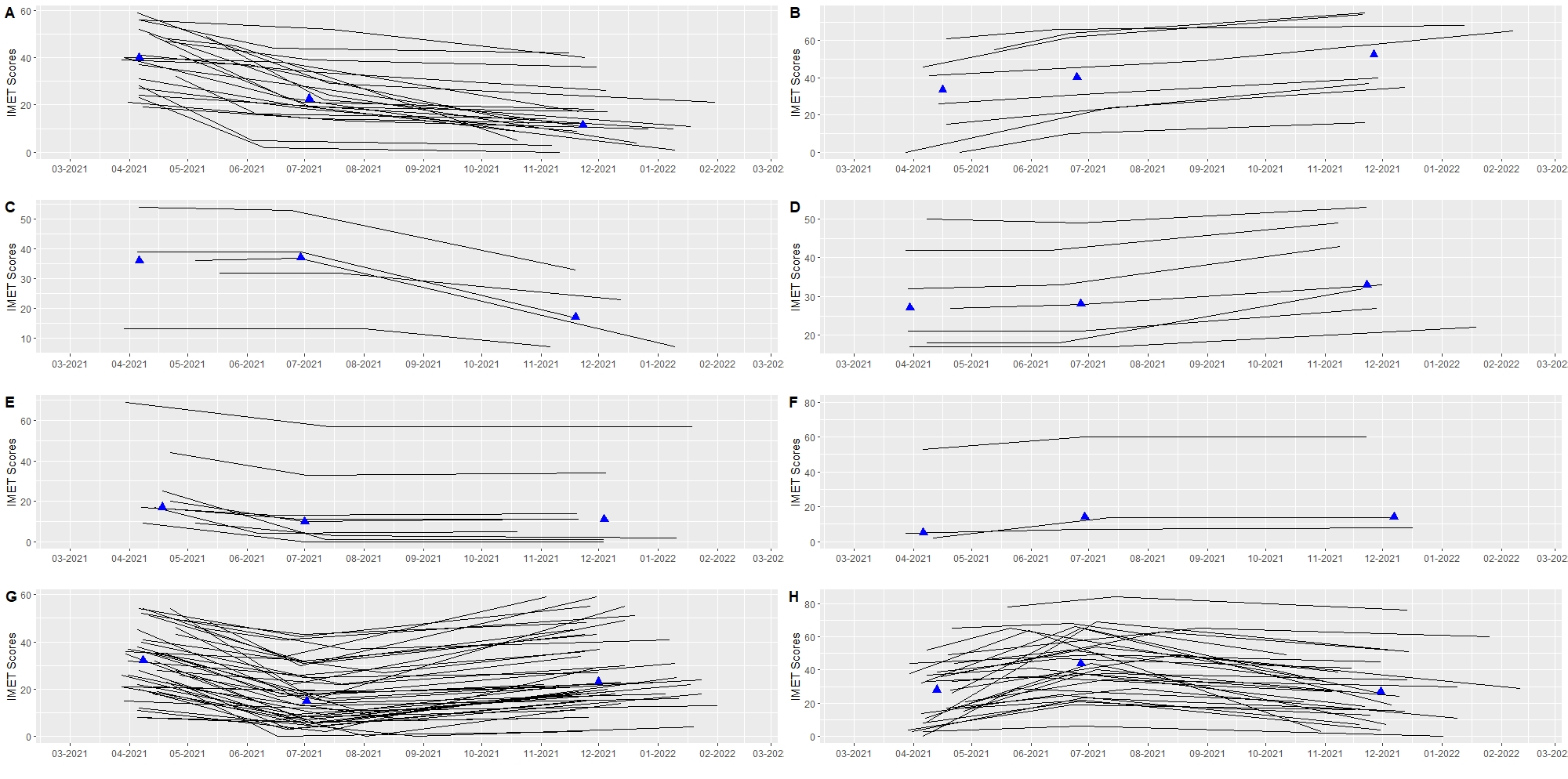


*Supplementary Figure 1: Individual courses of social participation*

*Blue triangle: median IMET Score with corresponding median date for each observed timepoint*

*A: T0-T1 fewer impairments, T1-T2 fewer impairments; n = 22*

*B: T0-T1 increased impairments, T1-T2 increased impairments; n = 8*

*C: T0-T1 consistent impairments, T1-T2 fewer impairments; n = 5*

*D: T0-T1 consistent impairments, T1-T2 increased impairments; n = 7*

*E: T0-T1 fewer impairments, T1-T2 consistent impairments; n = 9*

*F: T0-T1 increased impairments, T1-T2 consistent impairments; n = 3*

*G: T0-T1 fewer impairments, T1-T2 increased impairments; n = 43*

*H: T0-T1 increased impairments, T1-T2 fewer impairments; n = 29*

*
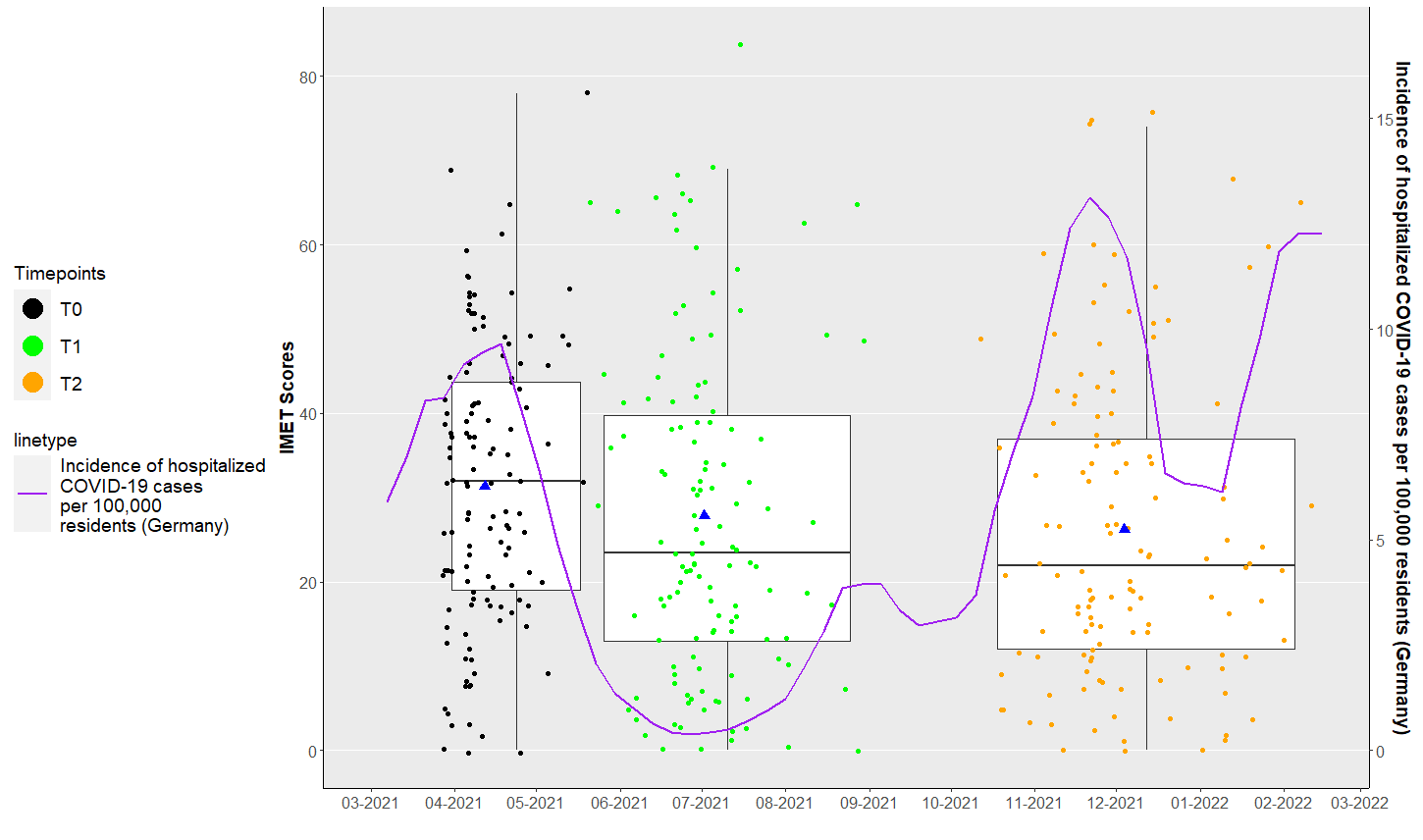
*

*Supplementary Figure 2: Median and mean change of social participation between T0, T1 and T2*

*blue triangle: mean IMET scores and date for each observation point; purple line: secondary Y-Axis (Incidence of hospitalization COVID-19 cases per 100.000 residents (Germany))*
